# Waist circumference vs body mass index in association with cardiorespiratory fitness in healthy men and women: a cross sectional analysis of 403 subjects

**DOI:** 10.1186/1475-2891-12-12

**Published:** 2013-01-15

**Authors:** Shiri Sherf Dagan, Shlomo Segev, Ilya Novikov, Rachel Dankner

**Affiliations:** 1 Department of Epidemiology and Preventive Medicine, School of Public Health, Sackler Faculty of Medicine, Tel Aviv University, Tel Aviv, Israel; 2 The Institute for Medical Screening, Sheba Medical Center, Tel Hashomer, Israel; 3 Unit for Biostatistics, The Gertner Institute, Sheba Medical Center, Tel Hashomer, Israel; 4 Unit for Cardiovascular Epidemiology, The Gertner Institute, Sheba Medical Center, Tel Hashomer, Israel

**Keywords:** Obesity indexes, Maximal exercise test, Periodic health examinations

## Abstract

**Objective:**

Body mass index (BMI) is more commonly used than waist circumference as a measure of adiposity in clinical and research settings. The purpose of this study was to compare the associations of BMI and waist circumference with cardiorespiratory fitness.

**Methods:**

In a cross-sectional study of 403 healthy men and women aged 50 ± 8.8 years, BMI and waist circumference were measured. Cardiorespiratory fitness was assessed from estimated maximal O_2_ uptake (VO_2_max), as calculated from a maximal fitness test.

**Results:**

Mean BMI (kg/m^2^) was 27.8 ± 3.7 and 25.5 ± 4.6; and mean waist circumference (cm) 94.1 ± 9.7 and 84.3 ± 10.4 for men and women, respectively. Both men and women reported an average of 2.5 hours of weekly sports related physical activity, and 18% were current smokers. Correlation coefficients between both BMI and waist circumference, and VO_2_max were statistically significant in men (r = −0.280 and r = −0.377, respectively, *p* > 0.05 for both) and in women (r = −0.514 and r = −0.491, respectively, *p* > 0.05 for both). In women, the contribution of BMI to the level of VO_2_max in a regression model was greater, while in men waist circumference contributed more to the final model. In these models, age, hours of training per week, and weekly caloric expenditure in sport activity, significantly associated with VO_2_max, while smoking did not.

**Conclusion:**

The differences observed between the sexes in the associations of BMI and waist circumference with VO_2_max support the clinical use of both obesity measures for assessment of cardiorespiratory fitness.

## Introduction

Obesity is a well-documented risk factor for morbidity and mortality; however, the association between body fat and pathology has not been fully elucidated. Though body mass index (BMI), calculated as weight in kilograms divided by height in meters squared, is the most common measure of obesity, it does not reflect body shape. Moreover, it can be misleading, such as in individuals with a high proportion of lean muscle mass. Waist circumference, a more accurate measure of the distribution of body fat [1], has been shown to be more strongly associated with morbidity and mortality [1-3]. Nevertheless, despite the American Heart Association’s recent endorsement of both BMI and waist circumference as primary tools for assessing adiposity [4], waist circumference is less commonly used than the BMI in both research and clinical settings. Associations between both BMI and waist circumference and disease risk factors have been shown to be sex dependent [5].

Increased physical fitness has been found to associate with reduced risk of cardiovascular mortality [6,7] and all-cause mortality [8]. By contributing to weight reduction and maintenance, physical activity also reduces morbidity and mortality risk indirectly. Recently, even incidental physical activity was shown to associate with cardiorespiratory fitness [9]. Increased BMI and waist circumference have both been associated with decreased cardiorespiratory fitness [10] and with obesity related metabolic abnormalities [11].

The purpose of the current study was to compare, in both men and women, associations of BMI and waist circumference, with cardiorespiratory fitness, as assessed by calculated VO_2_max, and to explore sex differences in these associations.

## Methods

This is a cross sectional study of 403 healthy subjects (222 men and 181 women), 25–65 years old, examined in the Periodic Examination Health Survey at the Sheba Medical Center in 2010. Subjects with chronic conditions that could interfere with physical activity: cardiovascular diseases, diabetes, stroke, malignancies, asthma, COPD, rheumatic diseases, or orthopedic problems, were excluded, as well as those on beta-blockers and other agents that would slow the heart rate and interfere with the fitness test.

After a brief explanation about the study and signing an informed consent form, each participant was measured by one surveyor for body weight, height, and waist circumference. Weight and height were measured on a digital medical scale, and waist circumference was measured three times at the level of the umbilicus. Subjects filled in the Periodic Examination Health Survey questionnaire, comprising information on: age, sex, smoking habits (by current smokers and non smokers), and routine sports related physical activity habits (number, duration, and type of physical activity training per week), and nutritional information (adherence to a certain diet, body weight changes over the previous year, and receiving of nutritional consulting). This information was reviewed for accuracy together with the examinee during a short personal interview conducted immediately before or after the fitness test by one interviewer. Weekly caloric expenditure for physical activity was calculated as the product of: the number of training sessions per week, duration of exercise, exercise intensity according to type of physical activity [12], and body weight.

For individuals who refused to participate in the study, unidentified data were extracted from medical files: age, sex, smoking habits, BMI (self-reported for height), and physical activity (yes/no) to check for selection bias of the study sample. The response rate was 94.3%; only 24 individuals refused to participate in the study. No significant differences were found in the distribution of age, smoking status, BMI and reported physical activity habits (yes/no) among respondents and those who refused to participate (not shown); however, the majority of those who refused were women (71%), a statistically higher proportion than men (*p* = 0.019).

All participants performed a maximal fitness test according to the Bruce protocol. They were required to achieve at least 85% of their age- predicted maximal heart rate (220 - age in years). VO_2_max was calculated according to the following formulas, in which “T” is the time (in minutes) spent on the treadmill according to the protocol [13,14]: In men: VO_2_max (ml/kg/min) = 14.76 - (1.379 × T) + (0.451 × T^2^) - (0.012 × T^3^); in women: VO_2_ max (ml/kg/min) = 4.38 × T - 3.9.

This study received the approval of the Institutional Review Board of the Sheba Medical Center.

### Statistical analysis

All statistical analyses were carried out using EXCEL and SPSS (versions 15). In the univariate analysis, Pearson’s correlation coefficients were calculated to assess the relationship between BMI and VO_2_max, between waist circumference and VO_2_max, and between BMI and waist circumference, in men and women separately, and for the study sample as a whole.

Sex specific multivariate linear regressions were performed in the “enter” method for men and women separately, and for the total sample as well. An adjusted multivariate linear regression model was calculated to examine the association between VO_2_max and BMI and between VO_2_max and waist circumference. Tests for detecting multicollinearity in the multivariate linear regressions were performed. We considered multicollinearity as a value of VIF ≥ 10 and/or tolerance ≤ 0.1 [15]. The interactions between obesity indexes and VO_2_max were tested in both sexes and described graphically. The Williams’ test was used to compare the correlation between BMI and VO_2_max and the correlation between waist circumference and VO_2_max in both sexes.

## Results

Participant characteristics and maximal fitness test results (VO_2_max) are presented in Table 1. Differences between the sexes were statistically significant for BMI, waist circumference, weekly caloric expenditure in physical activity, and VO_2_max. The Pearson’s correlation coefficient between BMI and VO_2_max was negative and statistically significant (*p* < 0.05) for both men: r = −0.280 and women: r = −0.514. The Pearson’s correlation coefficient between waist circumference and VO_2_max was also negative and statistically significant (*p* < 0.05) for both men: r = −0.377 and women: r = −0.491. The differences between the correlation of BMI and VO_2_max and the correlation of waist circumference and VO_2_max were significant in men (*p* < 0.001) but not in women (*p* = 0.45). The Pearson’s correlation coefficient between BMI and waist circumference was statistically significant (*p* < 0.05) for both men: r = 0.896 and women: r = 0.889.

**Table 1:** Characteristics (means ± SD) of 403 men and women in the study

**Variable**	**Women**	**Men**	**p**^**b**^
	**N = 181**	**N = 222**	
Age (years)	50.1 ± 8.7	49.7 ± 8.9	0.7
BMI (kg/m^2^)	25.5 ± 4.6	27.8 ± 3.7	<0.001
Waist circumference (cm)	84.3 ± 10.4	94.1 ± 9.7	<0.001
Hours of training per week	2.5 ± 3.1	2.5 ± 2.6	0.8
Weekly caloric expenditure on physical activity (kcal)	789.3 ± 820.1	1134.1 ± 1191.3	0.014
Cardiorespiratory fitness: VO_2_max (ml/kg/min)	39.2 ± 9.7	41.6 ± 8.7	0.007
Smoking (%)	18 ± 4	18 ± 4	0.9

Data are mean ± SD.

Abbreviations: BMI = Body Mass Index.

p values are for differences in the variables between women and men.

The statistical analyses performed: to compare the differences between men and woman for continuous variables t tests for independence groups were performed, for continuous variables that do not distribute normally the Mann–Whitney tests were performed and for the dichotomous variable smoking the Chi square test was performed.

In an adjusted multivariate linear regression model (not shown) that described VO_2_max according to waist circumference (without BMI), the variables: age, hours of training per week, and caloric expenditure in sport activity per week were found to associate with the level of cardiorespiratory fitness (*p* < 0.001 for all). Sex and smoking were not associated with VO_2_max (*p* = 0.214, *p* = 0.328, respectively), but the association between the interaction of waist circumference and sex, and between VO_2_max was of borderline statistical significance (*p* = 0.063), and thus sex was included in the final model.

In an adjusted multivariate linear regression model (not shown) that described VO_2_max according to BMI (without waist circumference), the variables: age, hours of training per week, and caloric expenditure in sport activity per week were found to associate with cardiorespiratory fitness (*p* < 0.001 in all). Here again, sex and smoking were not found to be significantly associated with VO_2_max (*p* = 0.16, *p* = 0.233, respectively). However, the association between the interaction of BMI and sex, and between VO_2_max was of borderline statistical significance (*p* = 0.057), and thus sex was included in the final model.

**Figure 1:**
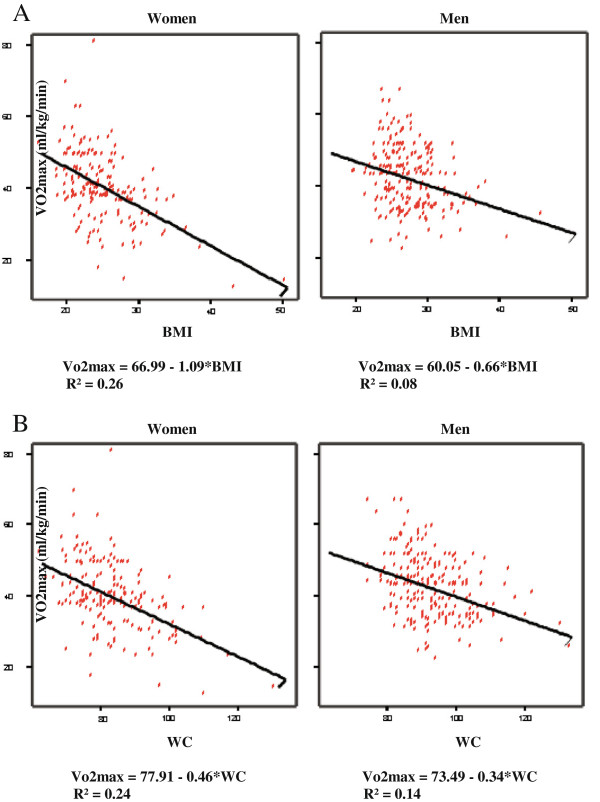
**The interaction between obesity indexes (BMI and waist circumference) and cardiorespiratory fitness, as measured by calculated VO**_**2**_**max. A: **The interaction between BMI and VO_2_max in men and women. For the for interaction between BMI*sex and VO_2_max in the adjusted multivariate linear regression models P = 0.057. Abbreviation: BMI = Body Mass Index. R^2^ - a measure of goodness-of-fit in linear regression. **B:** The interaction between waist circumference and VO_2_max in men and women. For the interaction between WC*sex and VO_2_max in the adjusted multivariate linear regression models P = 0.063. Abbreviation: WC = waist circumference. R^2^ - a measure of goodness-of-fit in linear regression.

Figure 1A and 1B illustrate the interaction of the two obesity indexes and sex in relation to VO_2_max. Figure 1A shows that the strength of the association between BMI and VO_2_max is greater in women than in men, yet the difference between the sexes is not constant, and is greater in the higher BMI values, and diminished in the lower BMI values. In men, for every 1 kg/m^2^ lower BMI the VO_2_max is 0.66 ml/kg/min higher, while in women, for every 1 kg/m^2^ lower BMI the VO_2_max is 1.09 ml/kg/min higher.

The graph of the interaction of waist circumference and sex in their association to VO_2_max (Figure 1B) shows that the strength of the association between waist circumference and VO_2_max is greater in women than in men, yet the difference between the sexes is not constant, and is greater in the higher waist circumference values, and diminished in the lower values. In men, with every 1 cm lower waist circumference the VO_2_max is 0.34 ml/kg/min higher, while in women, with every 1 cm lower waist circumference the VO_2_max is 0.46 ml/kg/min higher.

After ruling out multicolinearity between BMI and waist circumference both were entered into the linear regression models. Table 2 presents the adjusted multivariate linear regression model for the association of waist circumference, BMI and VO_2_max for men and women separately. In women, the model demonstrates that after adjusting for waist circumference, age, smoking status, number of weekly hours spent training, and caloric expenditure per week on physical exercise, for every 1 kg/m^2^ greater BMI, the VO_2_max is 0.94 ml/kg/min lower (*p* = 0.001). In men, the model demonstrates that after adjusting for BMI, age, smoking status, number of weekly hours spent training, and caloric expenditure per week on physical exercise, for every 1 cm greater waist circumference, the VO_2_max is 0.36 ml/kg/min lower (*p* = 0.001). Age, hours of training per week, and weekly caloric expenditure in physical activity were significantly associated with the cardiorespiratory fitness level for both men and women (Table 2).

**Table 2:** **The multivariate linear regression models for predicting cardiorespiratory fitness (VO**_**2**_**max) in men and women**

	**Reference category**	**Men**			**Women**		
		**N = 222; R**^**2**^ **= 0.453**			**N = 181; R**^**2**^ **= 0.404**		
**Variable**		Regression coefficient β	Standard error	P^a^	Regression coefficient β	Standard error	P^a^
Constant		84.10	5.08	<0.001	80.41	5.97	<0.001
BMI	1 kg/m^2^ increment	0.09	0.28	0.742	−0.94	0.28	0.001
WC	1 cm increment	−0.36	0.11	0.001	−0.13	0.12	0.29
Age	1 year increment	−0.30	0.05	<0.001	−0.19	0.07	0.006
Smoking	not smoking	1.23	1.19	0.301	0.09	1.50	0.952
Hours of training per week	1 hour/week increment	−2.18	0.91	0.017	−2.10	0.56	<0.001
Weekly caloric expenditure for physical activity	1 kcal/week increment	0.008	0.002	<0.001	0.011	0.002	<0.001

Abbreviations: BMI = Body Mass Index, WC = waist circumference.

p values are for association of the variables with VO_2_max.

R^2 ^- a measure of goodness-of-fit in linear regression.

The statistical analysis performed: multivariate linear regression models for predicting VO_2_max in men and women.

We note that for both men and women, smoking associated positively with cardiorespiratory fitness, though without any statistical significance (Table 2). The implication is that a person who smokes will have better cardiorespiratory fitness than a person who does not smoke, if all other variables are equal, including the amount of weekly training and caloric expenditure. We note also that increased weekly training associated negatively with cardiorespiratory fitness for both men and women. The implication is that a person who invests more time in physical activity per week will have poorer cardiorespiratory fitness than a person who invests less time, if all other variables are equal, including weekly caloric expenditure.

## Discussion

In this cross- sectional study associations between two obesity indexes (BMI and waist circumference) and between cardiorespiratory fitness, as measured by calculated VO_2_max, were both stronger in women than in men. For men, the correlation between waist circumference and VO_2_max was stronger, with statistical significance, than the correlation between BMI and VO_2_max. For women, while both correlations were higher than those for men, the correlation between BMI and VO_2_max was stronger than the correlation between waist circumference and VO_2_max, a difference not statistically significant.

Other studies have reported a wide range of values for correlations between BMI and VO_2_max [16-18], and between waist circumference and VO_2_max [16,17,19], with differences reported between the sexes. In a Finnish cross sectional study of 807 men and 633 women (age: 18–75 years) individuals within the same BMI category with normal waist circumference were more physically active and had better cardiorespiratory fitness than did those with high waist circumference [20]. Cardiorespiratory fitness was negatively associated with obesity, a relationship that remained after adjustment for level of physical activity. Physical activity levels were negatively associated with obesity in men, but not in women. In a cross sectional study that included young men only, the level of fitness was more closely associated with waist circumference than with BMI [21].

Similar to other findings [22], the correlations observed in the current study between waist circumference and BMI were high and statistically significant in both sexes. Nevertheless, the differences found between BMI and waist circumference concur with the distinction between these two measures of obesity, as highlighted by the United States National Health and Nutrition Examination Survey 1999–2004. There, when BMI was the measure of adiposity, 51.3% of overweight adults were metabolically healthy; conversely, 23.5% of normal weight adults were metabolically abnormal. In contrast, when waist circumference was the measure of adiposity, only 36.4% of abdominally obese adults were metabolically healthy, yet 28.3% of normal weight adults were metabolically abnormal [23]. Evidently, both adipose measures associate only partially, and differently, with metabolic normalcy.

In the current study, age, hours of training per week and caloric expenditure in sport activity per week, were highly associated with cardiorespiratory fitness in both men and women. Nevertheless, the linear regression models presented suggest that the preferred obesity index is different in men and women, and that BMI may better indicate cardiorespiratory fitness for women, and waist circumference for men. The R^2^ values for the statistical models of approximately 0.4 (Table 2) indicate that from simple and readily available characteristics (BMI, waist circumference, age, smoking status, number of weekly hours spent training, and caloric expenditure per week on physical exercise) cardiorespiratory fitness can be estimated quite well in a healthy population.

Analysis of the regression models showed VO_2_max to be associated positively with weekly caloric expenditure, yet negatively with the weekly number of hours of physical activity for the same caloric expenditure. The upshot is that, for the same caloric expenditure, engagement in more hours per week of physical activity is associated with a lower fitness level than engagement in fewer hours (i.e. at greater intensity). This applies to men and women, highlighting the importance of intensity of physical activity for maintenance of cardiorespiratory fitness for both sexes. We assume that similar investigation of athletes would reveal positive coefficients for both caloric expenditure and hours of activity, since their activity level is usually intense, and the level of intensity is not generally related to the duration of activity. According to a literature review, when total energy expenditure of exercise is held constant, exercise performed at vigorous intensity conveys greater cardioprotective benefit than exercise at moderate intensity [24].

The lack of a statistically significant association between smoking and cardiorespiratory fitness observed in the current study contrasts with a previous report of a negative association between smoking and physical fitness, as assessed by VO_2_max [25]. However, our lack of information on smoking history is a limitation that raises the possibility of reverse causality. Past smokers, classified here as nonsmokers, may have quit smoking due to a poorer health profile, and may thus show poorer physical fitness. Conversely, the current smokers may represent a selected subsample of the ‘healthier’ and more resilient study participants. Moreover, it is conceivable that had we divided the participants by the number of cigarettes smoked per day, we would have found an association between smoking and physical fitness. In addition, the relatively low proportion of smokers in the study sample, 18% for both men and women (compared with 28% and 13%, respectively, for the over age 20 year population estimated from nationwide surveys in Israel) [26], suggests that the lack of association could be due to small sample size or to reporting bias resulting from participants denying their smoking habits.

The differences we found between the sexes in associations of waist circumference with VO_2_max support the clinical use of this measure, in addition to BMI, for assessment of cardiorespiratory fitness.

Our findings support the stronger negative association observed in young adult men, between cardiorespiratory fitness and waist circumference, compared to BMI, in the Finnish Defense Force [21]. Encouraging ‘waist loss’ rather than weight loss alone has been shown to effectively motivate weight reduction [1]. Waist circumference measurement is rapid, inexpensive, and easily performed. However, despite the inclusion of waist circumference as a key diagnostic criterion for the metabolic syndrome [27,28], a uniformly accepted protocol for its measurement has not been established. Nevertheless, the high variability in location of measurement site [29] was not found to have considerable effect on associations of waist circumference with cardiovascular risk factors and mortality [29,30].

While waist circumference, and not BMI, reflects fat distribution, neither waist circumference nor BMI measures body tissue composition. Men and women differ considerably in fat proportion, as well as distribution. Sex-related differences, which are readily apparent in normal-weight men and women, may predispose to a spectrum of fat distribution phenotypes with obesity [31]. Higher prevalence of “apple” shaped obesity in men, ie. central obesity (“android obesity”) may explain the stronger relationship we observed between waist circumference and VO_2_max in men. In contrast, higher prevalence of “pear” shaped obesity, ie, more fat in subcutaneous areas, especially in the gluteal and femoral depots (“gynecoid obesity”), may explain the stronger relationship observed between BMI and VO_2_max in women. Sex-based differences in fat distribution may explain differences between the sexes in VO_2_max, as well as differences between obesity indexes. Proctor et al., found that when VO_2_max is normalized to kilograms of fat free mass, the sex difference in VO_2_max levels often disappears. They suggested expressing VO_2_max per unit of fat free mass when comparing the cardiorespiratory fitness of individuals with different body sizes and composition [32].

Participants of the current study are of Caucasian ethnicity. Thus, the current targets for BMI and waist circumference, which were derived from studies of predominantly white and European populations, were appropriate. However, the applicability of these targets to other populations has been questioned [33,34]. Further, contrary to BMI, waist circumference is independent of height. Even though waist circumference tends to be greater in tall adults, it increases at a “slower” relative rate than does height; short individuals would have greater absolute waist circumference-to-height ratios than would tall individuals. At present, studies examining circumference-height associations are inconsistent in their conclusions [35].

In this study we showed that associations between obesity and cardiorespiratory fitness are dependent on sex, and on the anthropometric measure used. Though a meta-analysis found BMI and waist circumference to be associated similarly to incident diabetes, most of the studies included did not analyze men and women separately, and some did not even adjust for sex [36]. A recent review reported a stronger association of measures of central obesity than BMI to diabetes, but similar associations to other cardiovascular risk factors, namely, hypertension and dyslipidemia [37]. However, the authors concluded that the cross-sectional design of the studies, as well as the lack of analysis by sex, limit generalizability of their conclusions. Since the participants of the current study were healthy men and women, we do not know if our findings apply to people with chronic illnesses.

Temporality of the relationship between obesity and physical fitness cannot be determined in the current study, due to its cross-sectional design. Reverse causality can therefore not be excluded, ie. people with poor physical fitness may gain weight and become more obese. The complexity of the relationship between physical fitness and obesity is further highlighted by reports of their differential effects on different diseases. In a systematic review, Fogelholm found the risk for all-cause and cardiovascular mortality to be lower in those with high BMI and good aerobic fitness than in those with normal BMI and poor fitness. In contrast, the concomitance of a high BMI with high physical activity level was associated with a greater risk for the incidence of type 2 diabetes and the prevalence of cardiovascular and diabetes risk factors than a concomitant normal BMI and low physical activity level [38].

The use of a calculated value for VO_2_max, rather than a direct measure, is a limitation of this study. The gold standard for measuring VO_2_max is by gas analysis during a maximal fitness test. However, this is an expensive test that requires highly skilled operators and motivated subjects. Since this test is not usually practical, formulas that predict VO_2_max have been developed over the years. VO_2_max can be evaluated by means of a fitness test or by other methods [39]. The Bruce protocol assumes that maximum oxygen consumption can be evaluated by the duration of time a subject is able to walk or run on a treadmill. The test score is the time taken for the test, in minutes, which can then be converted to an estimated VO_2_max score [13,14].

## Conclusions

In this study, both BMI and waist circumference were more strongly associated with VO_2_max in women than in men. In healthy men waist circumference correlated more strongly with physical fitness (as calculated by a maximal fitness test) than the BMI, whereas in healthy women BMI correlated somewhat more strongly with physical fitness than waist circumference. Our findings support previous ones of the need to measure waist circumference and not only BMI in clinical and research settings, as a means of better evaluating health status in both sexes. We emphasize the need to investigate men and women separately when studying obesity indexes and cardiorespiratory fitness.

## Abbreviations

BMI: Body mass index; VO_2_max: Maximal oxygen consumption; COPD: Chronic obstructive pulmonary disease.

## Competing interests

The authors declare that they have no competing interests.

## Authors’ contributions

SSD contributed to the design and conduct of the study, data collection and analysis, data interpretation and drafted the manuscript. SS contributed to the design and conduct of the study and to data collection. IN contributed to the design and data analysis, and to data interpretation. RD conceived of the study, and participated in its design and coordination, data analysis, data interpretation and helped to draft the manuscript. All authors read and approved the final manuscript.
